# Insights into the Structures and Multimeric Status of APOBEC Proteins Involved in Viral Restriction and Other Cellular Functions

**DOI:** 10.3390/v13030497

**Published:** 2021-03-17

**Authors:** Xiaojiang S. Chen

**Affiliations:** 1 Molecular and Computational Biology, Departments of Biological Sciences, Chemistry, University of Southern California, Los Angeles, CA 90089, USA; xiaojiac@usc.edu; Tel.: +1-213-740-5487; 2 Genetic, Molecular and Cellular Biology Program, Keck School of Medicine, Norris Comprehensive Cancer Center, University of Southern California, Los Angeles, CA 90089, USA; 3 Center of Excellence in NanoBiophysics/Structural Biology, University of Southern California, Los Angeles, CA 90089, USA

**Keywords:** structure, multimerization or oligomerization, viral restriction, mutation and cancer, innate and acquired immunity

## Abstract

**Apo**lipoprotein **B** mRNA **e**diting **c**atalytic polypeptide-like (APOBEC) proteins belong to a family of deaminase proteins that can catalyze the deamination of cytosine to uracil on single-stranded DNA or/and RNA. APOBEC proteins are involved in diverse biological functions, including adaptive and innate immunity, which are critical for restricting viral infection and endogenous retroelements. Dysregulation of their functions can cause undesired genomic mutations and RNA modification, leading to various associated diseases, such as hyper-IgM syndrome and cancer. This review focuses on the structural and biochemical data on the multimerization status of individual APOBECs and the associated functional implications. Many APOBECs form various multimeric complexes, and multimerization is an important way to regulate functions for some of these proteins at several levels, such as deaminase activity, protein stability, subcellular localization, protein storage and activation, virion packaging, and antiviral activity. The multimerization of some APOBECs is more complicated than others, due to the associated complex RNA binding modes.

## 1. Introduction

**Figure 1:**
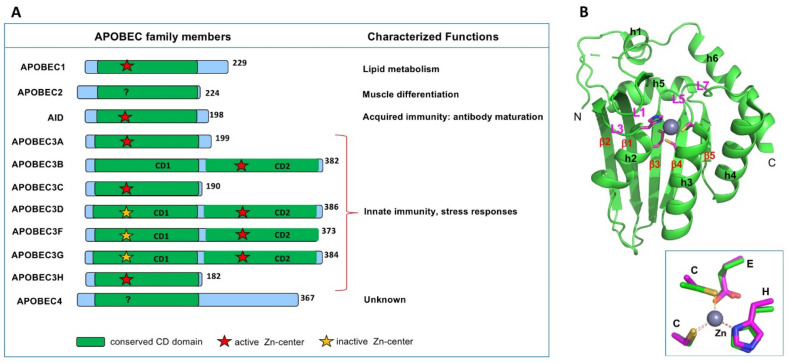
The apolipoprotein B mRNA editing catalytic polypeptide-like (APOBEC) family members in humans. (**A**) Members of the APOBEC family contain either one or two cytidine deaminase (CD) domains. Even though all CD domains contain the canonical Zn-center motif of **H-**[P/A/V]-E-X_[23–28]_-P-**C-**X2-4-**C** (x is any amino acid), some are catalytically active (indicated by a red star), and some are catalytically inactive (yellow star). Summary of the characterized biological functions of different members are shown on the right side. (**B**) The APOBEC2 (A2) core structure (PDBid 2nyt, containing residue 40–224)) showing the typical APOBEC core CD fold, which contains a five-stranded beta-sheet and six alpha-helices. Inset shows the highly conserved 3D arrangement of the Zn-center among all APOBECs.

There are eleven members of APOBEC (abbreviated from **apo**lipoprotein **B** mRNA **e**diting **c**atalytic polypeptide-like) proteins in humans (Figure 1A). All APOBEC proteins known so far contain a single or two consecutive conserved cytidine deaminase (CD) domains composed of an invariant five-stranded beta-sheet (β1–5) and six alpha-helices (h1–6) (Figure 1B) [1]. Each CD core structure of APOBECs contains a highly conserved spatial arrangement of the catalytic or pseudo-catalytic center residues consisting of an **H-**[P/A/V]-E-X_[23–28]_-P-**C-**X_2-4_-**C** motif (x is any amino acid) [2,3], where the H and two C residues coordinate a Zn atom, and the E residue is critical for polarizing a water molecule near the Zn-atom for catalysis (Figure 1B inset) [1].

Nine out of the eleven APOBEC members can catalytically deaminate cytosine to uracil, to cause C-to-U change on DNA or/and RNA. Remarkably, these APOBEC proteins utilize their common CD fold and C-to-U deamination activity, to execute diverse biological processes, including adaptive and innate immunity, lipid metabolism, muscle development, RNA editing, genetic mutations, and various associated diseases. Specifically, AID (activation-induced cytidine deaminase) is critical for the adaptive immune response in the antibody maturation to fight against foreign infectious pathogens (such as viruses) and non-infectious antigens [4,5]. In contrast, the seven APOBEC3 proteins (A3A, A3B, A3C, A3D, A3F, A3G, and A3H) are a crucial part of the innate immune system against various external viral pathogens, as well as endogenous retroviral elements [6,7,8,9,10,11,12,13,14,15]. However, non-immune-related functions are also linked to some APOBEC functions. APOBEC1 (A1), the first identified APOBEC and the APOBEC family’s namesake, plays an important role in lipid uptake and cholesterol metabolism [16,17]. APOBEC2 (A2) is shown to function in cardiac and skeletal muscle development [18,19,20,21,22]; APOBEC4 (A4), which is reported to be capable of enhancing transcription from a broad spectrum of viral and cellular promoters [23], is expressed in testis with its biological function yet to be determined [24]. In addition, dysregulated APOBEC deaminase activity can cause mutations of genomic DNA or RNA and lead to genetic diseases and cancer (see References [25,26,27] and references therein).

Many APOBEC proteins form various multimeric or oligomeric complexes [28,29,30,31,32], and multimerization appears to impact the functions of APOBEC at different levels. These multimeric complexes are also consistent with the purification of APOBEC proteins from cell lysates, where APOBECs often exist as heterogeneous complexes even in the presence of reducing agents that can reduce potential disulfide bonds between free cysteines. These complexes contain various cellular RNAs, suggesting a role of RNA binding in forming these large APOBEC complexes [32,33,34]. Despite the common involvement of RNA binding in the large multimer formation, the initial step of multimerization of APOBECs appears to form a stable dimer, instead of the initial trimer or higher oligomers [29,35,36,37,38,39].

Even though studies over the past decades have revealed a great deal of APOBEC functions, there are still many interesting questions to be addressed as to the detailed mechanisms of multimerization and their exact roles in regulating functions. Recently published structural and biochemical data of APOBEC proteins have helped to address some of the questions relating to multimerization and function for some APOBEC members.

To review the structures and functions of the APOBEC protein family, we focus on the structural and relevant biochemical data on the multimerization status of all structurally characterized APOBECs and the associated functional implications. Since the publication of the first APOBEC structure in 2007, many full-length or partial domain structures of APOBEC proteins have been determined by crystallography and NMR, and more recently by Cryo-EM. A3D and A4 are the only two members with no experimental structure determined so far. The available data indicate that different APOBEC members multimerize with different styles and mechanisms. In the following sections, we discuss the multimeric status of all the APOBEC members with available structures deposited in the databank, regardless of whether an individual APOBEC member has antiviral activity or not. The discussion order starts from single-domain APOBECs (AID, A1, A2, A3A, A3C, and A3H) and then double-domain A3s, using A3G as an example.

## 2. Activation-Induced Cytidine Deaminase (AID)

AID is essential for the antibody maturation in the adaptive immune response to neutralize infectious pathogens and non-infectious antigens. Thus, AID is a critical enzyme for the acquired immunity to fight against viruses through either natural infection or vaccination. The immune functional role of AID is dated back to bony fish [2,40]. During antibody maturation, immunoglobulin (Ig) genes undergo somatic hypermutation (SHM) and class switch recombination (CSR), both of which are initiated by AID deaminase activity on the Ig gene DNA [41,42,43,44,45]. AID mutates Ig genes specifically at the variable (V) region for SHM and the switch (S) region for CSR. For SHM on the V region, AID targets a hotspot motif WRCH (W = A/T, R = A/G, and H = A/C/T) for deamination [43,44]. For CSR, the targeted S-regions in mammals are enriched in the AGCT sequence, a palindromic motif of WRCH, and deamination at both strands can lead to double-strand breaks (DSBs) to initiate antibody class switching from IgM to other isotypes [46,47,48]. Patients with AID mutations that disrupt AID deaminase activity have impaired CSR and/or SHM and impaired antibody response that leads to prolonged infection, a syndrome known as hyper-IgM immunodeficiency [1,49,50]. AID biochemistry was recently reviewed [51].

Five crystal structures of AID from two different constructs have been reported [52,53], both of which lack the C-terminal 15 residues needed for CSR and contain other mutations or deletions to generate soluble AID monomeric proteins in solution. One structural form (represented by 5W0R) [52] is from a less mutated construct containing residues 6–181 out of the full-length 198 amino acids. The other structural form is from the more mutated version (5JJ4) [53] with multiple mutations and one internal deletion within the first 36 residues to mimic the N-terminal sequence of another APOBEC A3A and ends at residue 183. The wild-type AID protein is in a large heterogeneous multimeric form that contains bound-RNA species from the cells, which is mostly present in the insoluble pellet fraction of *E. coli* expressing human AID cell lysates. RNase A treatment of cell lysates containing the wild-type AID is required to generated soluble heterogeneous multimeric complexes and is also needed to activate the deaminase activity of AID [54], indicating that RNA binds to AID and blocks catalysis. How AID binds cellular RNA and what is the functional multimeric status of AID in cells, or if AID forms a complex with other potential cellular proteins for a particular function, requires further investigation.

A critical question for AID function is, what are the mechanisms of inhibition of its deaminase activity on genomic DNA outside the Ig gene? Would RNA binding serve this role in some way? It remains unclear regarding the mechanism of AID targeting the Ig gene for C-to-U mutation for SMH and the CSR of antibodies in activated B cells. Such specific Ig gene targeting may be tightly controlled at multiple levels, to prevent accidental mutations of the genome. An open chromatin for active transcription upon B cell activation is a known prerequisite for the targeting [55]. The hotspot DNA sequence motif (WRCH) for deamination provides another level of specificity [42,56]. For S-region targeting by AID in CSR, evidence suggests the high density of G-repeat sequences (G-quadruplex or G4) within the S-region with a high binding affinity to AID may play a role in the specific targeting [57]. G4 structured substrates are shown to have strong preferential binding to AID through a bifurcated substrate-binding surface and induce AID cooperative oligomerization, which is proposed to be an AID-targeting mechanism to S-regions through the G-repeat DNA sequences [52]. There is also a proposal that cellular proteins or RNAs may play a role in actively recruiting AID to target Ig genes in activated B cells [57,58,59,60,61]. More studies are needed to reveal if AID needs to form larger complexes with other proteins or RNAs for regulation and the specific targeting of Ig genes but not to other genes that are also actively transcribed in B cells.

## 3. APOBEC1 (A1)

There is no reported evidence that A1 can restrict viral infection to date. A1 is discovered initially as a specific mRNA editor for regulating lipid uptake in the liver and small intestine in mammals [16,62]. A1 is essential for editing apolipoprotein B mRNA (termed ApoB-RNA for simplicity) at a specific site, resulting in C to U mutation to create an early stop codon and produce a shorter ApoB48 protein from the non-edited ApoB100 [17,63,64]. This editing of ApoB-RNA regulates the levels of low-density lipoprotein (LDL) that is an atherogenic disease risk factor. The A1 editing activity on ApoB-RNA was later shown to require a protein cofactor, APOBEC1 complementation factor (ACF or now called A1CF) [65,66,67,68]. More recently, another cofactor, RNA binding motif protein 47 (RBM47), has been identified. Both A1CF and RBM47 interact with A1 in order to edit RNA [68,69,70,71], and A1/A1CF or A1/RBM47 is the minimal complex reconstituted in vitro to show editing activity on ApoB-RNA [65,66,67,68,72]. Different A1/cofactor complexes were recently shown to have editing activity on multiple RNA targets, including ApoB-RNA [69,72,73,74].

Active A1/cofactor complex was isolated from the nucleus as high-molecular-weight aggregations called 27S editosome [75,76]. One possible function of the 27S editosome aggregates may be to provide a subcellular location that can offer coordinated RNA-editing in the nucleus and subsequent safe export of the edited RNAs to the cytosol. However, it remains unclear about the oligomeric status of A1 and how A1 interacts with its cofactors within the 27S editosome complex. The crystal structure of A1 alone has been solved to 3.5 Å resolution [77], which reveals a unique dimerization mode among APOBECs. The dimer form of A1 can be detected by using biochemical and cellular methods [17,78,79,80]; thus, the dimer interaction is expected to be relevant to the regulation of certain functions of A1 inside cells, even though the exact biological relevance of this dimerization has yet to be determined.

**Figure 2:**
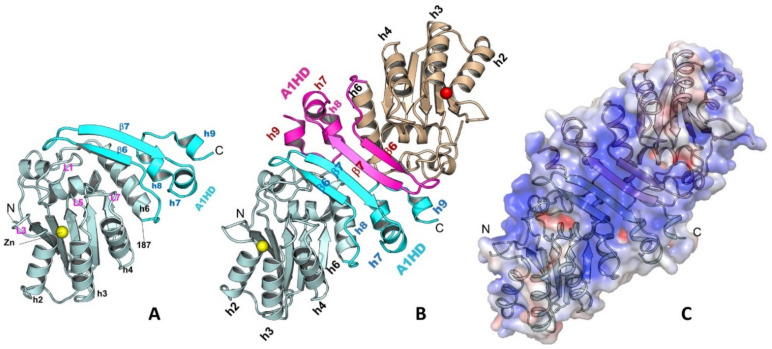
The structure of A1 monomer (**A**), dimer (**B**), and the surface charge feature of a dimer (**C**) (PDBid 6X91). A1 has a unique C-terminal domain A1HD (A1 hydrophobic domain) that mediates the protein-protein dimer formation. A long strip of positively charged surface is formed through dimerization, which may be used for recruiting nucleic acids or other protein partners.

The A1 dimerization is mediated through its unique C-terminal hydrophobic domain (A1HD)(Figure 2A,B) [77]. The N-terminal core deaminase domain of A1 (residues 15–187) has the typical APOBEC CD fold [1,81], while its C-terminal A1HD (residues 188–236) structure contains a β-hairpin (β6 and β7) and three small helices (h7, h8, and h9) (Figure 2A). The A1 dimerization interface via this A1HD has an extensively buried surface area of 1526.5 Å^2^, which involves mainly hydrophobic interactions at the core and hydrogen bonds at the peripheral. A point mutation at the interface, L173Q, can disrupt the dimer into a stable monomer. The dimer formation revealed by the high-resolution structure is consistent with several reported biochemical and functional studies [17,78,79,80]. The detailed dimerization interactions in the A1 crystal structure can explain many of the previous mutational data. Mutations of the C-terminal hydrophobic residues, such as L135F, F156L, and L189F [82], were shown to disrupt dimerization, which is likely due to the disruption of folding and destabilizing the structure near the C-terminus, as these residues are buried inside the structure. Additional mutations of the hydrophobic residues around this region, such as L182A and I185A, were found to disrupt RNA editing activity [78], likely due to the disruption of the hydrophobic packing between the A1HD and its core deaminase domain.

Despite of unknown functional relevance of dimerization, the C-terminal A1HD is important for A1 catalytic function. The A1 dimer structure reveals a large positively-charged surface that spans across the two paired β-hairpins of A1HDs, branching out to the two Zn-active centers near the N-termini (Figure 2C). Mutations on this positively charged patch significantly affected RNA binding and editing [78,82,83,84]. Deleting the C-terminus up to residue 196 to remove most of the β-hairpin structure motif of A1HD and much of this positively-charged surface showed a major reduction in RNA editing activity [78]. Further deletion of the C-terminus up to residue 188 (C∆48) resulted in diminished deaminase activity on both RNA and DNA, suggesting a role of A1HD in regulating A1 activity on RNA and DNA deamination, possibly by preventing non-specific aggregation resulted from the extra-hydrophobic residues at the C-terminal h6 of the core deaminase domain. The reduction of catalytic activity by the mutations/deletions around the A1HD may not be directly related to the disruption of dimer formation. The point mutation abolishing the dimer formation (A1 L173Q) still showed significant deaminase activity on RNA in the presence of a cofactor [77], suggesting neither the cofactor binding nor deamination require the dimer formation.

A possible role of A1 dimerization may be related to regulating protein aggregation/solubility in the large 27S editosome in the nucleus. The surface of h6 of the A1 core structure is much more hydrophobic than that of any other APOBECs. The hydrophobic h6 and the immediate C-terminal A1HD have multiple solvent-exposed hydrophobic residues as in a monomer. Dimerization effectively shields most of these surface-exposed hydrophobic residues of A1, which can reduce/eliminate random aggregation through these hydrophobic residues. Considering the importance of A1HD in possibly interacting with RNA directly, A1 dimerization and binding to a cofactor (A1CF or RBM47) during RNA editing may be mutually exclusive events. It is worth noting that the two known A1 cofactors A1CF and RBM47 have sequence and biochemical features that closely resemble the large family of proteins commonly associated with the biologically relevant phase-separated aggregates or liquid-liquid phase separation (LLPS) inside cells (to be published results) [85,86,87,88]. Thus, it is intriguing to posit that the aggregation of purified A1 cofactors seen in vitro may be relevant to the observed 27S large complex as a form of phase-separated aggregates that could play a role in regulating storage, subcellular localization, and activity of A1 editosomes in a similar manner as the LLPS aggregates reported for many other systems. The detailed oligomeric status of A1 in the large editosome complex and the interaction of A1 with A1CF/RBM47 and its target RNA are worthy of future investigation.

## 4. APOBEC2 (A2)

A2, together with AID, are the two most ancient APOBEC members, and its gene is dated back to jawed vertebrates and bony fish [2,40]. However, no immune function in restricting viruses has been discovered for A2. Even though it has the typical Zn-center components and 3D conformation as other APOBECs, the catalytic activity of A2 has not been demonstrated yet [1,89,90], which could be due to lack of deaminase activity or due to missing cofactors or unidentified substrates. Despite the lack of observed deaminase activity of human A2, studies of A2 in different experimental animal models suggest important roles in muscle development [19,20,22], retina regeneration [91], and left-right axis specification [92].

**Figure 3:**
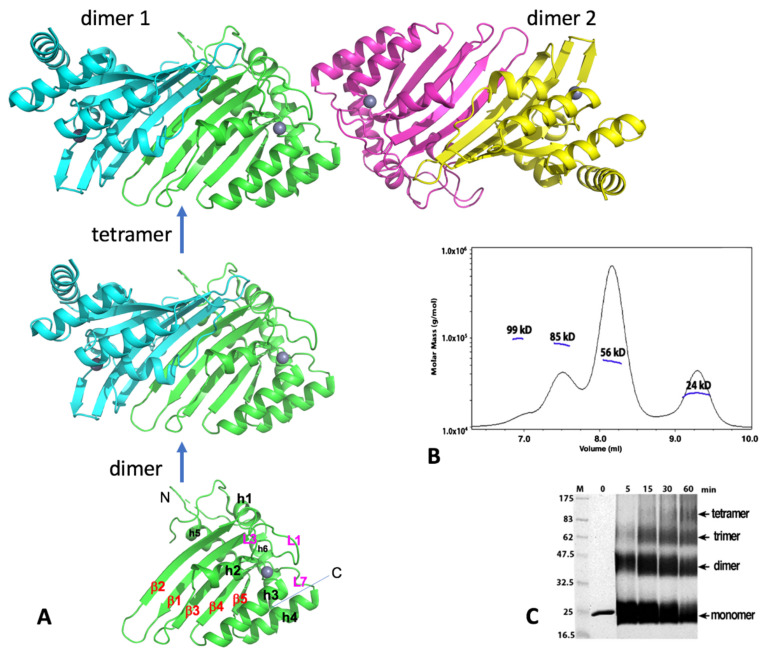
Human A2 crystal structure. (**A**) The crystal structure of A2-core tetramer, dimer, and monomer (PDBid 2nyt, residues 40–224). (**B**) Molecular weights from size-exclusion chromatography coupled with multi-angle light dynamic scattering (SEC-MALS) data for the A2 (full-length, 26 kD for a monomer) at a protein concentration of 1 mg/mL. Four peaks were observed, corresponding to the approximate molecular weight of a monomer (24 kD), dimer (56 kD), trimer (85 kD), and tetramer (95 kD). The dimer species is the predominant form. (**C**) Time course of glutaraldehyde cross-linking with the A2 protein. Reactions with A2 (2 µg total protein) were performed for the indicated time, with 0.25% glutaraldehyde at room temperature, 25 mM HEPES (pH 7.0), 160 mM NaCl, and 10% glycerol; they were then quenched with 1 M Tris, pH 8.5, with 2X SDS loading buffer and run on a 12% SDS-PAGE gel for 70 min, at 200 V. Proteins were visualized by Coomassie staining. Cross-linking reveals four bands, corresponding to a monomer, dimer, trimer, and tetramer band, respectively. Monomer and dimer appear to be the predominant forms under this cross-linking condition. Both full-length (residues 1–224) and the N-terminal truncated A2-core (residues 40–224) showed similar results. Note: Panels B and C are to-be-published data.

The crystal structure of A2, which contains the core CD domain of wild-type A2 sequence but missing the highly negatively charged N-terminal 40 residues (A2-core), reveals a tetramer composed of two identical dimers that come together in a head-to-head (or N-to-N) mode (Figure 3A). This crystallized A2-core also dimerizes uniquely by pairing of two long β-strands (β2) of two subunits, which expands the five-stranded β-sheet of a subunit sideways with a near two-fold symmetry (Figure 3A) [1]. Publication of the A2 crystal structure raised an interesting question whether any other members of APOBEC may also use the same dimerization mechanism observed in the A2-core structure. The available data discussed in later sections reveal that each of the dimer-forming APOBECs has its own unique dimerization interface.

Two dimers interact with each other to form a tetramer through head-to-head interactions that involve the helix 1 (h1), loop 1 (L1), loop 7 (L7), and helix 6 (h6), all of which are around the Zn-center pocket. Such tetramerization interactions around these Zn-center loops lead to the closed Zn-center pockets (thus inaccessible) for the two subunits at the tetramer interface, leaving the two subunits on the outer ends of the tetramer with their Zn-center pockets being open and accessible for nucleic acid binding. Results of size exclusion chromatography coupled with multi-angle light dynamic scattering (SEC-MALS) assay or cross-linking of the purified full-length or the N-terminal truncated A2 proteins, using glutaraldehyde were also consistent with the formation of dimer and tetramer (Figure 3B,C; see legends for conditions), even though a minor trimer species is also observed. The SEC-MALS assay shows a similar monomer, dimer, trimer, and tetramer trend, with the dimer being the predominant form under the assay condition. While the observed monomer, dimer, and tetramer are easy to understand, but the trimer is puzzling. One possibility could be a preformed dimer interacting with a monomer.

An NMR structure of a truncated version of mouse A2 (PDB id: 2RPZ deposited as “to be published”) was determined as a monomer form. Full-length human A2 was later also characterized as monomeric form, using NMR and biochemical methods in a buffer containing 50 mM NaCl at a protein concentration between 0.5 and 2 mg/mL [93]. Based on the NMR characterization, it was proposed that the flexible N-terminal 40 residues of A2 are tumbling near the dimerization interface to inhibit the dimerization in solution [93]. While the biologically relevant oligomeric form still needs further verification, the potential explanation for the observed discrepancy in the oligomeric status of A2 could be due to a few factors, such as differences in assay methods, in buffer conditions, or/and protein concentrations. Higher salt concentration (160 mM) and 10% glycerol were used in the SEC-MALS and cross-linking assays (see Figure 3B,C legends). Taken together, these results suggest that A2 can exist as a monomer, dimer, trimer, and tetramer in purified form. Unlike most other APOBEC members, A2 is well-behaved during purification, likely because A2 does not bind to cellular RNAs. Despite the structural and biochemical data available to date, the functional oligomeric forms of A2 or whether A2 needs to form complexes with other cellular proteins for function requires further investigation.

## 5. APOBEC3A (A3A)

**Figure 4:**
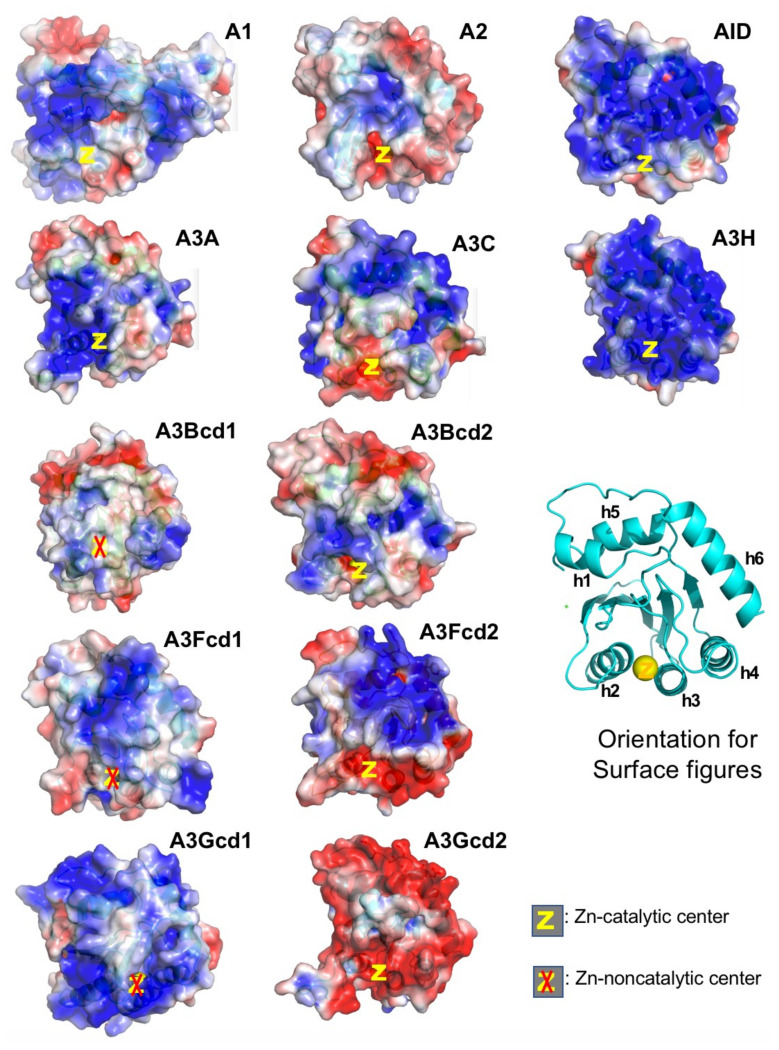
Surface charge of APOBECs CD domains. The general orientation for all panels is shown as the ribbon structure. Among all catalytic CD domains (not including CD1 of A3s), only A3A and A3H have their Zn-center embedded within positively charged surfaces (PCS, in blue). A2 and A3Gcd2 have the least PCS among single-domain and double-domain APOBECs, respectively. It is worth noting that A2, A3Gcd2, and A3A are the only three proteins that do not form large RNA-bound multimeric forms in cell lysates (data not shown). Note: A3Fcd1 structure is to be published structure. PDBids for other structures are A1, 6X91; A2-core, 2nyt; AID, 5W1C; A3A, 5KEG; A3C, 3VOW; A3H, 5W3V; A3Bcd1, 5TKM; A3Bcd2, 5CQI; A3Fcd2, 3WUS; A3Gcd1, 5K83; A3Gcd2, 3IQS.

A3A belongs to the seven-member APOBEC3 (A3) subfamily that shows activity in restricting RNA and DNA viral infections. A3 subfamily includes three single-domain (A3A, A3C, and A3H) and four double-domain (A3B, A3D, A3F, and A3G) APOBECs. A3A and A3H are shown to have the highest catalytic activity in converting C-to-U and mC-to-T on ssDNA among all APOBEC proteins [89,94,95]. One possible explanation for the much higher catalytic activity for A3A and A3H may be that these are the only two APOBECs with their catalytically active Zn-center being surrounded by positively charged area and thus can efficiently bind the ssDNA substrate directly to the Zn-pocket (Figure 4). The A3A is thought to play a role in restricting foreign infectious DNA viruses [96,97,98,99,100] and in restricting internal retroelements [101,102,103]. However, elevated expression or/and defective regulation of A3A can also lead to mutations of genomic DNA and contribute to various diseases, including cancer [100,104,105,106]. More recently, A3A is also shown to deaminate RNA substrates bearing specific structural features [107,108], and editing of RNA by A3A is shown to occur in tumor cells [109].

Interestingly, despite having a strong positively charged surface, A3A does not form large RNA-bound multimeric complexes as A1, AID, A3H, and other double-domain full-length A3s, suggesting that having a highly positively charged surface does not necessarily mean strong RNA-binding and formation of RNA-bound multimeric complexes. As a result, A3A can be relatively easily purified without RNase treatment from cell lysates, and purified A3A has a high level of deaminase activity on ssDNA regardless of RNase A treatment or not. However, A3A can also deaminate RNA with certain sequence/structure features [108,109], suggesting A3A can specifically bind certain types of RNA to the Zn-active center for deamination, but not for multimerization.

**Figure 5:**
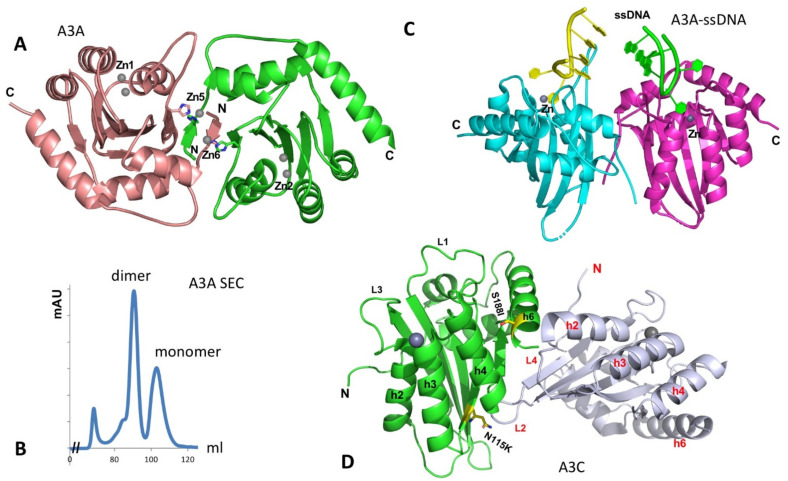
The structure of A3A and the two packed A3C molecules in the crystal asymmetric unit. (**A**) The dimer structure of A3A without binding ssDNA (PDBid: 4XXO). The dimerization is mediated mainly through the N-terminal arm exchange between two subunits (colored in brown and green), which is further cemented by two Zn-atoms (Zn5, Zn6) coordinated by residues from two subunits. The Zn1 and Zn2 are active site Zn-atoms. (**B**) The SEC assay of wildtype A3A purified from *E. coli* expression system on Superdex 75 at a protein concentration of 2 mg/mL, showing monomer and dimer peaks in a buffer containing 20 mM Tris-HCl, 250 mM NaCl (pH 8.0), 1 mM DTT, and 1 mM EDTA (to be published data). The small peak at the left side is void volume (aggregated A3A protein). No RNA can be detected in any of the peaks. (**C**) The structure of ssDNA-bound A3A as monomer (DPBid: 5KEG, 5SWW) that is superimposed on to the two subunits of the apo dimer structure shown in (A). This superimposition does not show additional interaction of the bound ssDNA at the two Zn-center of each monomer with any residues from the other monomer, suggesting the short ssDNA bound to the active center does not need a dimer. However, these structural data alone cannot exclude the possibility that longer ssDNA binding to one Zn-center can bind to the other monomer to enhance the binding. (**D**) The crystal structure of A3C (PDBid: 3VOW), showing two A3C molecules packed into one asymmetric unit, which has the largest intermolecular contact interface in the crystal packing. The positions of the S188I variant (S188 side chain) and N115K (N115 side chain) are shown in sticks.

The structure of the A3A monomer in solution was first solved by NMR [110]. A crystal structure of A3A was later determined as a dimer [111]. The A3A dimer interaction is mediated by protein-protein contacts that are cemented by the coordination of two Zn atoms involving the N-terminal residues of the two subunits (Figure 5A) [111]. The characterization of the oligomeric form of purified A3A was reported to be mostly monomeric, using dynamic light scattering (DLS) [112,113]. On our hand, the purified A3A exists in stable dimer and monomer forms on Superdex 75 SEC assay in the presence of reducing agent (Figure 5B, to be published), possibly due to higher protein concentration. However, when two co-crystal structures of A3A with ssDNA have been determined by two groups independently, both A3A-ssDNA complex structures are in monomeric form [114,115]. The two co-crystal structures reveal nearly the same substrate ssDNA interaction mode, i.e., a monomeric A3A Zn-active site interacts mainly with 3 nt of a kinked ssDNA centered around the target C, explaining the preferred substrate tri-nucleotide motif for deamination.

Hill coefficient of ~2 of binding ssDNA is observed for ssDNA containing one C residue on an inactive A3A mutant [111]. Mutation of two residues H11 and H56 that is expected to disrupt the dimer interface showed a mixed result, with H11A showing about a 10-fold reduction but H56A having no significant change in binding affinity [111], and there is no obvious cooperativity of ssDNA binding by the wild-type A3A [111,114]. Binding assay also shows that a dimer of A3A is not required for binding ssDNA [114], which is consistent with the co-crystal structure of a monomeric A3A binding to ssDNA [114,115] and also with the monomeric form suggested from an in-cell study, using molecular brightness analysis [32]. If superimposing the two monomeric A3A-ssDNA structures on the apo-A3A dimer structure, there is no clash between the bound ssDNA, but there are no additional interactions with the ssDNA if binding as a dimer because the distance between the two ssDNAs bound to the two Zn-centers are too far away from each other to make additional contacts, suggesting that A3A dimerization may not impact the binding to the short ssDNA (Figure 5C). However, given the highly active deaminase activities of A3A on ssDNA and RNA, it still remains to be seen if the apo-A3A dimer has a regulatory role or whether A3A interacts with cellular proteins to form functional complex for regulation of A3A activity, subcellular localization or/and turnover rate.

## 6. APOBEC3C (A3C)

Two polymorphic versions of A3C are discovered in human with a single amino acid change at position 188, S188, and I188 [116]. The more prevalent version is A3C S188 (or A3C) found in approximately 98% population. Human A3C expresses high levels in CD4+ lymphocytes and can be efficiently packaged into HIV virions [116,117,118,119]. However, A3C has very weak anti-HIV activity [116,117,120,121]. Nonetheless, A3C can be targeted by HIV Vif and E3-ligase for degradation [122,123]. A3C is also reported to have a broad antiviral activity for several retroviruses and DNA viruses, such as restricting the replication of simian immunodeficiency virus from African green monkey (SIVagm) and rhesus macaque (SIVmac), murine leukemia virus (MLV), endogenous retrotransposons LINE-1 (L1), and reducing the infectivity of herpes simplex virus (HSV), human papillomaviruses (HPV), Bet-deficient foamy virus, and hepatitis B virus (HBV) [98,117,118,124,125,126,127,128,129,130,131].

Purified A3C is in monomeric form only in high salt buffer containing 0.5 M arginine [122], or at low protein concentration without arginine [116,132]. Purified A3C forms large aggregates in the absence of arginine (unpublished data), suggesting a tendency to self-oligomerize. In addition, A3C forms large heterogeneous RNA-bound multimer complexes in cell lysates if RNase A is not included in the lysis buffer, indicating its strong RNA-binding propensity. This is consistent with the observation for its efficient virion packaging, which is an RNA-binding-dependent process.

Two crystal structures of A3C (3VOW and 3VM8) from the same crystal form have been determined to different resolutions [122]. Even though considered monomeric, the structure has a relatively large interface between two molecules packed into one asymmetric unit (ASU) in the crystal cell (Figure 5D). However, the two molecule interaction is not a two-fold symmetric contact. Thus, propagation of such interactions is expected to lead to “-head-tail-head-tail-“ polymers. This crystal packing interface within the asymmetric unit was not considered a biologically relevant dimer interface giving the monomeric form in solution [122].

Interestingly, the A3C I188 variant (or S188I to show the change from Ser to Ile) was later found in approximately 2% of the total population or about 10% of people of African descent based on the available genetic data. This S188I variant displayed 5- to 10-fold higher anti-HIV activity than the prevalent A3C (S188) [116,132,133]. This higher anti-HIV activity of S188I was attributed to the gained ability to form dimer, which is supported by the evidence from size exclusion chromatography (SEC) and cross-linking experiments [116,132]. This notion is further strengthened by the similarly enhanced anti-HIV activity of a forced tandem dimer through linking two A3C or two S188I variants together [116,132,134]. These results indicate that forced dimerization is sufficient to significantly increase the anti-HIV activity regardless of the A3C S188 or the I188 variant at position 188 [116,132,134]. This significantly enhanced anti-HIV activity cannot be accounted for by the slight increase of deaminase activity and the virion package level, which highlights the importance of dimerization of A3C in restricting HIV. One plausible explanation for this ~10-fold enhancement of anti-HIV activity is that dimerization through S188I mutation, or forced linker may gain efficient scanning of ssDNA or processivity [116,132]. However, an A3C double-mutant with its WE residues on loop 1 replaced with RK has significantly increased deaminase activity without enhancing dimerization, and the mutant has enhanced its restriction activity against HIV and LINE-1 replication to a level similar to that of A3G [135], a much greater enhancement than the S188I variants or its forced dimer, suggesting that the deaminase-dependent restriction activity alone can also dominate the antiviral activity in this A3C mutant.

While it is unclear why and how the A3C I188 variant dimerizes while A3C (S188) is a monomer in solution, it was thought that the dimer interface might be somewhat similar to the A3C crystal packing interface in an ASU [116,122,132] (Figure 5D). This thought is based on the close proximity of S188I and residue N115K to the crystal packing interface and the enhanced dimerization for these mutants [122,132]. Here, S188I does not directly participate in dimerization interaction but may alter the conformation around it to affect the dimer interface interaction (Figure 5D). N115K is facing toward the ASU packing interface, and a lysine residue is expected to add hydrogen bonds to strengthen the interface. Considering A3C multimerization associated with RNA-binding and its tendency to aggregate in the absence of arginine, it remains interesting to learn if and how A3C and A3C S188I dimerizes in the presence and absence of nucleic acids or in a cellular environment. Additionally, if the prevalent A3C (S188) version loses its potency in anti-HIV and anti-retroelements by mutating I188 to S188 during evolution [116,133], such a loss of function in restricting HIV and retroelements raises an intriguing question regarding whether A3C has gained any new biological functions outside anti-retroviral activity.

## 7. APOBEC3H (A3H)

A3H in humans is the most polymorphic APOBEC member [136,137], with seven distinct haplotypes (hap I–VII) and four alternatively spliced variants ((SVs) SV154, SV182, SV183, and SV200) being identified in human cells [136,138,139]. These naturally occurring A3H variations identified in different individuals are shown to have different deaminase activity, subcellular localization, in vivo stability, and anti-HIV activity [37,38,39,95,136,138,139,140,141,142]. Among the four catalytically active haplotypes, hap II, V, and VII, have over 20- to 40-fold higher deaminase activity than hap I by in vitro assays [95]. Even though A3H hap I (A3H-I) can be stably expressed and purified from *E. coli* for in vitro assays, it was reported that A3H-I is not very stable in mammalian cells and is mostly distributed to the nucleus, and thus most relevant to genomic mutation in cancer [143,144]. A3H hap II (or A3H hereafter) is the most common version in humans and also has potent anti-HIV and retroelement activity [138,140,141,142,145,146]. Furthermore, similar to A3A, A3H hap I, II, V, and VII are all highly active in deaminating not only C but also methylated C (mC) on DNA [89,94,95], and the biological relevance of the hyper-catalytic activity on mC is unclear to date.

High-molecular-weight (HMW) complexes of the wild-type A3H similar to those of A3G from mammalian cells are the predominant multimerization forms [28,29,39,147]. These HMW complexes of A3H have a molecular weight range roughly in the range of 500 kDa or larger as they come out near the void volume peak in the size column chromatography on Superdex-200 [141]. RNase A treatment can reduce the HMW to low-molecular-weight (LMW, in the range of 30–100 kDa) complexes and activate the deaminase activity of A3H [39], indicating RNA-binding mediated multimerization and deaminase-inhibition of A3H in cells. Wild-type A3H in *E. coli* cell lysates also forms large multimeric complexes with bound RNAs. RNase A treatment of the lysates can convert the multimeric form to a dimeric A3H form containing stably bound dsRNA, which is well protected from further RNase A digestion [36,37,38]. High-salt (2 M NaCl) treatment can then dissociate the dsRNA to convert the dimeric A3H into monomeric form [39]. Stable monomeric A3H can also be obtained by point mutations H114A or W115A/C116S, which no longer forms large multimers in cell lysates because of the disruption of the RNA binding required for dimer formation [38,39].

**Figure 6:**
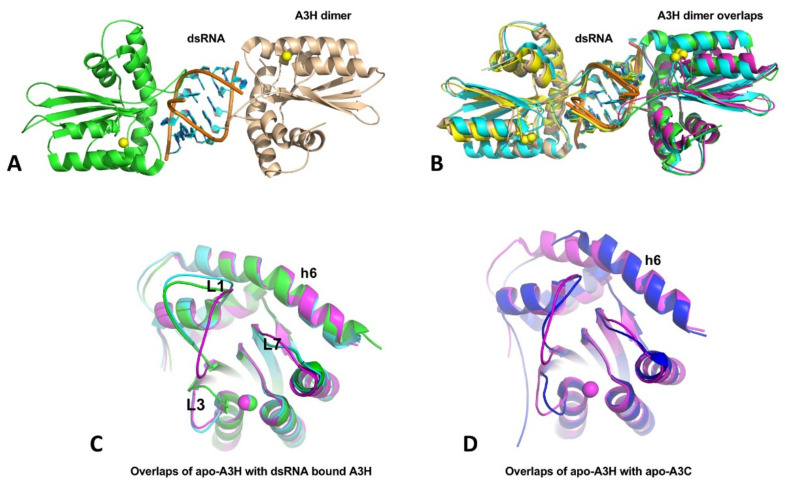
The crystal structures of A3H and dimer formation through dsRNA binding. (**A**) The A3H dimer (5W3V) with two subunits being connected through a dsRNA bound in between. No protein-protein contact exists between the two subunits within a dimer. (**B**) Superimposition of three available A3H dimers from three different organisms (5W3V, 6BBO, 5Z98), showing highly conserved dimerization mechanisms. (**C**) Superimposition of apo-A3H monomer structure with one subunit from the A3H dimers (5W45, 5W3V, and 5Z98), showing that conformational differences only for loops 1, 3, and 7, which are directly involved in dsRNA binding. (**D**) Superimposition of apo-A3H (5W45) and apo-A3C (3VOW) reveal that A3H has two extra alpha-helical turns for h6, compared to A3C and all other known APOBEC structures.

A3H structures have been published from four groups within a short period [36,37,38,39], with three being dimeric A3H from human and two monkey species, and one being a monomeric mutant of human A3H. Even though the three A3H dimers are from different primates, their structures are all highly conserved, and all contain a dsRNA that bonds two A3H subunits together (Figure 6A,B). In this dimer form, both A3H subunits interact directly with the dsRNA mainly through loops 1, 3, 7, and h6, with no direct protein–protein contacts in the dimer. Although all three A3H dimers are purified from *E. coli* cells, the dsRNA region of all three structures contains seven base pairs (5w3v, 5z98, and 6BBO) [36,37,38]. Two of the three structures (5w3v and 5z98) have a resolution sufficiently high to reveal nearly identical dsRNA sequences that match several *E. coli* genomic locations [37]. The human genome should have multiple matching sequences with the dsRNA sequences, the biological significance of binding to these particular dsRNA sequences requires future investigation. The human A3H mutant W115A/C116S is crystallized as a monomer free of bound RNA (5w45) [39]. The core structure of the RNA-free monomer is essentially the same as that of the three RNA-bound dimers, with the loops showing different conformations, including loops 1, 3, and 7 that interact with RNA (Figure 6C), suggesting RNA-binding does not induce major conformational changes. However, the A3H core CD structure has an h6 that is about two alpha-helical turns (seven amino acids) longer than all other APOBEC structures determined so far (Figure 6D).

The question is how RNA binding produces HMW complexes while the dsRNA-bound A3H is only a dimer? The dsRNA bound in the dimeric A3H is buried between two subunits and well-protected from RNase A digestion (Figure 6A,B). It is likely that, in the HMW complexes, ssRNA overhangs extending from the dsRNA 5′ and 3′ ends can interact with other areas of the same A3H dimer or another A3H dimer, and the propagation of such interactions with other A3H dimers may lead to RNA-mediated multimerization of A3H in the form of HMW complexes. This scenario suggests A3H can bind RNA outside the areas interacting with dsRNA. If so, the two A3H mutants W115A/C116S and H114A that are purified as RNA-free monomers should still retain some RNA binding capability. Indeed, RNA binding assay demonstrated that A3H mutants W115A/C116S and H114A still have comparable binding affinity to long (50 nt) ssRNA or ssDNA oligomers but show significantly reduced binding affinity for short (13 and 8 nt) ssDNA oligomers, confirming general non-specific RNA binding capability outside the dsRNA binding region [39]. However, stable large oligomer formation of A3H needs both dsRNA binding for dimerization and other non-specific RNA binding for larger complex formation.

RNA binding to A3H inhibits deaminase activity, and RNase A treatment can mitigate the inhibition, which likely is attributed to the digestion of the ssRNA parts outside the RNA-resistant dsRNA region. Interestingly, isolated dsRNA-bound A3H dimer faction is catalytically active on ssDNA [39]. Because of the technical difficulties in assessing the RNA-binding status during catalysis on ssDNA, it remains to be evaluated whether the RNase A-resistant dsRNA bound to loops 1 and 7 allow access to the Zn-active center by the ssDNA substrate. Given the highly stable RNA-bound A3H dimer, RNase A treatment of A3H in cell lysates possibly digests away the ssRNA parts, leading to the removal of ssRNA that blocks ssDNA substrate binding and its access to the Zn-center pocket. Indeed, the HMW fractions show little deaminase activity but become highly active after RNase A treatment that converted the HMW to LMW species [39].

While RNase A-sensitive ssRNA bound to the non-dsRNA binding region can effectively block deaminase activity, several insights into potential roles of dsRNA binding of A3H can be summarized from the recent structure/function studies [36,37,38,39,148]. First, mutants disrupting the dsRNA binding and A3H dimerization can become hyperactive in ssDNA deamination, suggesting dsRNA binding can reduce the accessibility of the Zn-active site and, thus, dampened the deaminase activity as a dimer. Second, the dsRNA-binding defective A3H mutants show a shift of subcellular localization from the cytosol to the nucleus, generating genomic mutations with the elevated deaminase activity. Third, some of these RNA binding mutants become less stable in cells, indicating a role in enhancing the stability of A3H through dimerization. Forth, mutants defective in dsRNA binding are also defective in packaging into HIV virion and restricting HIV replication. Whether these four types of biological consequences associated with dsRNA binding can also be linked to ssRNA binding will need further investigation. Some residues may play a role in binding to only dsRNA or ssRNA or ssDNA, while other residues could participate in binding to both RNA and substrate ssDNA. For example, R26 on A3H loop 1 is shown to be involved in RNA binding, ssDNA substrate interaction, and deaminase activity [148].

## 8. Double-Domain A3s

All four double-domain APOBECs (A3B, A3D, A3F, and A3G) are shown to form large and heterogeneous multimeric complexes in cell lysates and in situ fluorescence study [28,29,30,31,32,147,149]. Three out of the four double-domain A3s (A3D, A3F, and A3G) have strong activities in restricting HIV and endogenous retroelements [3,9,10,15,123,150]. Even though the exact biological functions of A3B are still not fully characterized, it is shown to play a role in DNA replication stress and crisis response [151,152,153], and deregulation of its expression can lead to cancer mutation and perhaps promote evolution or programmed cell death [3,26,27,154,155,156]. These APOBEC3 proteins all have a tendency to bind RNA. However, compared with single-domain APOBECs (such as AID and A3H), RNA binding modes and the functional consequences associated with RNA binding are more complicated for double-domain A3s, which remains poorly understood. The RNA binding ability by four double-domain A3s is important for the encapsidation into HIV virions [157,158,159,160,161,162]. For A3G, RNA binding plays an important role in the multimerization process, and RNase A digestion alone can convert HMW to LMW form in cell lysates. For A3B and A3F, however, RNA binding appears to impact the large multimeric form differently. Despite the presence of RNA in the HMW complex of A3B and A3F, and RNase A treatment can elevate their deaminase activity significantly, RNase A treatment alone in buffers containing reducing agent does not convert its HMW to LMW complexes for A3B [163] and A3F [164], suggesting protein-protein interactions also play a role in the large multimeric formation of A3B and A3F.

A3G arguably shows the most potent anti-HIV and anti-retroelement activities among all APOBEC proteins [9,10]. Only full-length A3G containing both the N-terminal CD1 and C-terminal CD2 is shown to have antiviral activity. One of the major functional requirements for the anti-HIV activity of A3G is its ability to bind RNA. However, it is clear that not all RNA binding is equal for its anti-HIV activity [35,147,158,160,165,166,167]. Emerging evidence start to suggest how RNA is bound to A3G in terms of locations and binding affinity and possibly what type of RNA is bound by A3G in terms of RNA sequence and structural features may determine the functional outcome of A3G [147].

A3G appears to have a clear division of labor in function; the RNA binding ability of A3G is endowed by its catalytically inactive CD1. In contrast, RNA or DNA binding by the catalytic CD2 alone cannot be detected by using gel shift assay and rotational anisotropy [168], but it can be detected by cross-linking reagents [167,169], suggesting a very week interaction. However, by cleverly designing mutations around the active pocket to enhance ssDNA binding, co-crystal structures of an ssDNA substrate or a dinucleotide binding to CD2 have been obtained [170,171]. CD1 binds various cellular RNAs and is essential for virion packaging and for anti-HIV and anti-retroelement through an RNA binding-dependent mechanism [172,173,174,175]. CD1 can enhance the deamination efficiency of CD2 by two or three orders of magnitude and critical for the processivity of full-length A3G [168,172,176], which likely is due to its ability to coordinate ssDNA binding instead of RNA binding. Even though RNA binding is through CD1, the deaminase activity catalyzed by the Zn-active center of CD2 is still blocked by RNA binding as RNase A treatment is required for the activation of the deaminase activity of full-length A3G but not CD2 alone [149,168].

For the structural studies of double-domain A3s, a divide-and-conquer approach has been employed in the past, to study individual CD1 or CD2 domains, due to the strong tendency to aggregate for the wild-type full-length proteins. The CD2 domains of A3B [177,178], A3F [179,180,181,182], and A3G [168,183,184,185,186,187] have been determined alone, using X-ray and NMR, and in complex with ssDNA, using crystallography [115,170,188,189,190] or A3F-CD2 with Vif/CBFb partners, using cryoEM [191], all as monomeric form. The CD1 of A3B [163] and A3G [149,192] has been determined by X-ray or NMR, which offers some useful information about RNA binding and multimerization for the full-length A3G and A3B. The progress in the individual domain structural studies over the years has led to the eventual determination of two recent A3G full-length structures [147,171]. Here we focus on our current understanding of the full-length A3G structures, which begin to shed more light on the possible mechanisms of RNA binding by A3G and the associated multimerization and biological functions.

**Figure 7:**
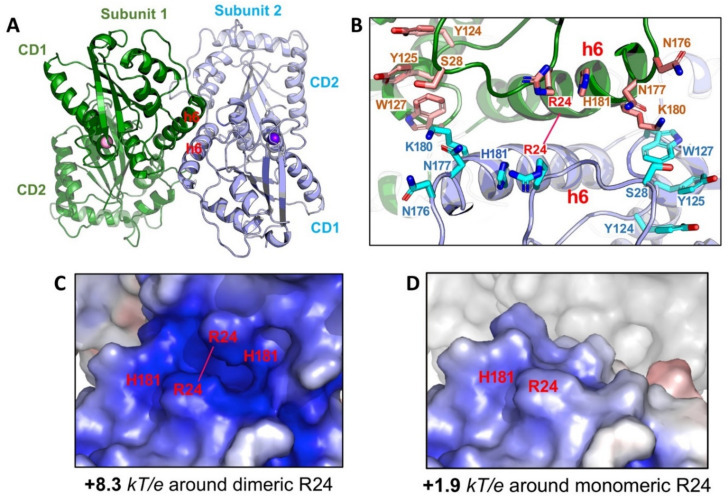
Structure of full-length rA3G. (**A**) The rA3G dimer structure (6P3X). Dimerization is mediated through CD1-CD1 contacts centered around helix 6 (h6) of CD1. (**B**) The positively charged residues centered around R24 on both sides of the dimer junction. Dimerization brings these positively charged residues from each subunit to close proximity. (**C**) The closely positioned charged residues around the dimer junction result in significantly enhanced positively charged electrostatic potentials (PEP or diPEP), as shown by the blue surface (+8.3 kT/e). (**D**) The surface charge potentials of a monomer at the same area around R24, revealing much less positively charged electrostatic potentials (+1.9 kT/e), as compared to those of a dimer.

How is the multimeric A3G formed from the newly synthesized molecule in the cell? The crystal structures of a full-length A3G from a rhesus monkey (*Macaca mulatta*, rA3G) provides a structural basis for the initial nucleation in the multimerization process [147]. One of the less mutated rA3G constructs (6P3X, containing a total of 6 residue mutations) forms a dimer through N-terminal CD1-to-CD1 contacts (Figure 7A). This CD1-to-CD1 dimer contact of the full-length rA3G is essentially the same as that observed for the rA3G-CD1 domain alone [149], and the CD1-CD1 contact configuration is similar to the N-to-N of the dimer-dimer interactions of A2 tetramer structure [1]. The dimer formation and the residues involved in the protein-protein dimer interactions of A3G, such as R24, F126, and W127, are also supported by multiple biochemical studies [35,176,193,194,195,196].

The full-length A3G structures solved from two groups show three different orientations between CD1 and CD2, indicating that CD1 and CD2 interfaces can differ depending on the orientation and that CD1-CD2 is connected by a flexible linker [147,171], which is consistent with a simulation study [197]. The interesting questions raised from this observation are: How many relatively stable orientations between CD1 and CD2 are there for A3G? Why can the conformation with a certain orientation can be captured? What are the functional relevance of each of these CD1-CD2 orientations? One explanation for these different conformations with different CD1-CD2 orientations based on the structural information is that one conformation (as in 6P3X) may be for RNA binding and dimerization/multimerization, while another conformation (as in 6WMA) may be for substrate ssDNA binding and deamination [147,171]. However, at this point, more data will be needed to address these fascinating questions about the functional relationship of domain orientation of A3G, which could be relevant to other double-domain APOBEC members as well.

In the dimeric rA3G mutant structures [147], no strong electron density for bound RNA can be discerned despite detectible residual RNA in the purified protein as assayed by RNA gel, suggesting that the residual RNA bound to rA3G is heterogeneous in sequence or binding locations or binding occupancy. It was first noticed many years ago that newly synthesized A3G in cells could stay as lower molecular weight as a monomer and dimer for a short time, and then it can multimerize into large RNA-bound HMW (or HMM) within 30 min [198]. The dependence of A3G multimerization on RNA binding has clearly been demonstrated as RNase A treatment of cell lysates expressing A3G reduced the HMW complexes to low-molecular-weight (LMW) species within the molecular weight range of tetramer, dimer, and monomer [193,198] and also by mutations [35,147,149,166,199].

The fact that extensive RNase A treatment can convert HMW to LMW form down to monomer in addition to dimer, and higher multimers also suggests that RNA-binding may stabilize rA3G dimer observed in the crystal structure [35,149,199]. Multiple positively charged residues align on either side of the rA3G dimeric interface centered around R24 (Figure 7B) [147,149]. Positioning of these positively charged residues closely to each other through dimerization significantly enhances the dimer-junction positive electrostatic potentials (diPEP) (Figure 7C,D), suggesting possible RNA binding through this diPEP. Mutation disrupting both the rA3G protein-protein dimerization interface and RNA binding to the enhanced diPEP area resulted in clean monomeric rA3G without RNase A treatment [147], which suggests that the protein-protein interactions and the RNA binding to the enhanced diPEP area can reinforce each other in forming a stable dimer. This RNA-bound dimer may serve as the nucleation for forming larger multimeric forms, a process possibly requiring additional non-specific RNA-binding to outside the diPEP areas of rA3G. In this regard, the multimerization process of rA3G from an initial dimer is similar to that of A3H, even though no direct protein-protein interaction exists in the A3H dimer [36,37,38].

While RNA binding to the diPEP area plays a role in reinforcing rA3G dimerization, it is not required for its virion encapsidation as mutation disrupting RNA binding to diPEP still retains a significant level of HIV restriction [147]. The RNA binding to the positively charged region around h2 of CD1 (h2 patch) is also not required for virion encapsidation and anti-HIV activity. Interestingly, mutations on the diPEP surface resulted in loss of 80–90% of deaminase activity, and mutations on both diPEP and h2 positively charged patch on CD1 resulted in undetectable deaminase activity under the assay conditions. However, these mutations all retained about 70% of wild-type HIV restriction activity [147], indicating that these RNA binding areas are not required for deaminase-independent anti-HIV activity [11,166,174,200,201]. Alternatively, these areas of CD1 can, in addition to binding RNA, serve additional functions necessary for deaminase activity, such as binding ssDNA substrate or regulating inter-CD1/CD2 domain orientation, to enhance CD2 deaminase activity. However, the RNA binding through loop 7 residue W127 of A3G-CD1 is essential for virion packaging and thus for HIV restriction [166,202,203,204,205,206]. Thus, similar to A3H, there are different RNA binding modes for rA3G through different areas to exert distinct functions, including regulation of deaminase activity, multimerization, virion packing, inhibition of reverse transcription, and restriction of HIV infection. The different RNA binding mechanisms and the associated functions of A3G appear to be much more intricately intertwined than originally thought. A good grasp of its complexity will require further structural and functional studies of A3G and other double-domain A3s in the future.

## 9. Summary

Multiple structures of full-length proteins or truncated domains of APOBEC proteins have been determined, to date, by crystallography, NMR, and Cryo-EM. The accumulated structural and biochemical data reveal that, despite a common core CD fold and a conserved Zn-center that can bind ssDNA/RNA polymer as the substrate, each APOBEC member shows different multimerization status. Among them, the structures of AID and A3C are crystallized as monomers, even though higher multimeric forms for functions cannot be excluded. All other five APOBECs (A1, A2, A3A, A3H, and A3G) with known structures are shown to form dimers that can potentially act as the building block for higher multimeric forms. However, the dimerization modes of these APOBECs are unique for each member. While the biological relevance of dimerization is still unclear for A1, A2, and A3A, the dimerizations of A3H and A3G are shown to have a number of functional roles. Clearly, not all RNA binding is equal for A3H and A3G (and possibly other double-domain A3s), suggesting different RNA-binding modes for different functions. Future structural and functional studies of APOBECs and their interactions with various nucleic acids and other cellular components will be needed to understand the regulations of APOBECs proteins through binding to RNAs and other cellular partners.

## Data Availability

Not applicable.

## Abbreviations

A1CF	APOBEC1 complementation factor
A1HD	APOBEC1 C-terminal hydrophobic domain
ASU	asymmetric unit
CD	cytidine deaminase
CSR	class switch recombination
diPEP	dimer-junction positive electrostatic potentials
HMW	high molecular weight
LLPS	liquid-liquid phase separation
RBM47	RNA binding motif protein 47
LMW	low molecular weight
SEC-MALS	size-exclusion chromatography coupled with multi-angle light dynamic scattering
SHM	somatic hypermutation
